# Executive Function Improvement in Normal Pressure Hydrocephalus Following Shunt Surgery

**DOI:** 10.3233/BEN-2009-0249

**Published:** 2009-12-07

**Authors:** Ezequiel Gleichgerrcht, Andrés Cervio, Jorge Salvat, Anselmo Rodríguez Loffredo, Luciana Vita, María Roca, Teresa Torralva, Facundo Manes

**Affiliations:** ^1^Institute of Cognitive Neurology (INECO)Buenos AiresArgentina; ^2^Raúl Carrea InstituteBuenos AiresArgentina; ^3^Institute of NeuroscienceFavaloro UniversityBuenos Aires Argentina

**Keywords:** Normal pressure hydrocephalus, ventriculoperitoneal shunt, neuropsychological testing, executive functions, cognitive recovery

## Abstract

The aim of this investigation was to evaluate improvement of executive functions after shunt surgery in patients with early normal pressure hydrocephalus (NPH). Patients with NPH were assessed before and after shunt surgery with tests shown to be sensitive to damage to the prefrontal cortex (PFC). Significant differences were found between basal and follow-up performances on the Boston Naming Test, the backwards digits span, Part B of the Trail Making Test, and the number of words produced on the phonological fluency task. In conclusion, our study reveals that patients with NPH who respond positively to continuous slow lumbar cerebral spinal fluid drainage and receive a ventriculoperitoneal shunt implant, improve their performance on tasks of executive function. Due to the high demand for this form of mental processing in real-life complex scenarios, and based on the severe executive deficits present in both demented and non-demented NPH patients, we encourage the assessment of executive functions in this clinical group.

## 1. Introduction

Since its first description by Hakim and Adams in 1965, normal pressure hydrocephalus (NPH) has been characterized by a clinical triad of cognitive impairment, gait disturbance, and urinary incontinence, together with enlarged ventricles but normal pressure of the cerebrospinal fluid (CSF) [1,2]. While onset of gait abnormality usually precedes the other clinical signs and develops as the most prominent symptom [3,4] cognitive decline can become severely impairing, causing confusion and disorientation, apathy, and motor slowing [5].

Similarities of the gait pattern between NPH and Parkinson's disease, including short steps, leg rigidity, and impaired postural reflexes [6], shed light on the subcortical nature of the disorder, also characteristic of the cognitive deficits observed. For example, patients are impaired on tasks of memory recall, but show preserved memory storage on recognition tasks [7]. In addition, there is evidence of frontal lobe dysfunction, usually evidenced through changes in personality [8]. In particular, patients suffering from normal pressure hydrocephalus show severe executive dysfunctions, including difficulties to think abstractly, and inability to organize, plan, and adapt current and past knowledge to alter future behavior. These deficits are also present in non-demented patients with hydrocephalus, who besides showing intact global functioning, normal verbal intelligence, and no signs of clinical depression, exhibit significant executive impairment [9].

ISSN 0953-4180/09/$17.00 © 2009 – IOS Press and the authors. All rights reserved

Even though it may only account for up to 5% of all cases of dementia [7], studying the underlying bases of cognitive decline in NPH is important because recovery occurs in some patients after removal of CSF, which may be successfully achieved by implanting a ventriculoperitoneal (VP) shunt [10]. Gait improvement seems to occur in most cases [11], but cognitive decline is the least likely symptom of the triad to recover [12]. Indeed, studies comparing cognitive performance before and after VP shunt have shown contradictory results. While some authors report little or no postoperative improvement [13–15] others have found cognitive recovery in more than 65% of shunted patients [12].

Most authors have found improvement of memory functions [10,12,16–19], and psychomotor speed [16, 18,19], but little evidence has been reported concerning recovery of executive functions. Consistently, a strong correlation has been reported between positive response to continuous CSF drainage and gait recovery [7,12], but few studies have looked at cognitive recovery prospectively [16]. For these reasons, the aim of our study was to compare cognitive performance before and after the insertion of a VP shunt in a group of patients with clinical response to continuous CSF drainage. More specifically, we aimed at investigating whether tasks of executive function, specifically designed to detect damage to the prefrontal cortex, would show improvement after such surgical procedure.

## 2. Materials and methods

### 2.1. Patients

Patients were recruited from our clinic according to the following criteria: 1) cognitive impairment; 2) gait disturbance and/or urinary incontinence; 3) magnetic resonance imaging showing ventriculomegaly (*Evan's index* $> 0.3$); 4) dependence on activities of daily living; 5) unknown cause for the hydrocephalus; 6) no history of cardiovascular or psychiatric disorders; 7) no history of previous shunt surgery. Ten patients met inclusion criteria, all of which gave their informed consent prior to entering this study.

### 2.2. Procedure

Patients were initially assessed with a neurological examination of gate and urinary incontinence, and a standard neuropsychological battery. To determine candidacy for surgery, patients were monitored for CSF pressure levels during 48 hours, after which they were subjected to slow lumbar CSF drainage (150 ml/day) for three consecutive days. Post-drainage improvement on any of the triad symptoms was determined with an abbreviated version of our battery. Eligible patients were implanted a Codman Hakim Programmable Valve (Codman Corporation, Raynam, MA), and reassessed six to eight months after surgery.

Pre- and post-operative gait assessment included (1) speed performance on a 20 meter track; (2) clinician's subjective impression of gait change; and (3) the Hauser Ambulatory Index (AI), which assesses functional gait capacity [20]. Urinary incontinence was determined using the Urinary Incontinence Quality of Life Scale (I-QOL), a 22-item questionnaire concerning frequency and urgency of urinary incontinence [21]. The neuropsychological battery assessed: (1) general cognitive status, with the Mini Mental State Examination (MMSE) [22], and the Addenbrooke's Cognitive Examination (ACE) [23]; (2) language, with the adapted version of the Boston Naming Test for naming [24], the adapted version of the Token Test for comprehension [25], and listing of animals in 60 seconds for semantic fluency [5]; (3) attention, with the forward digit span task of the Weschler Memory Scale – Revised (WMS-R) [26], and the Trail Making Test Part A (TMT-A) [27]; (4) executive function, with the backward digit span task [26], phonological fluency (words starting with letter "P" in 60 seconds) [5], Part B of the Trail Making Test (TMT-B) [27], and the Frontal Assessment Battery (FAB) [28]. When appropriate, alternative versions of the tests included in the preoperative battery where used in follow-up assessment to minimize practice effects, employing different stimuli for the attention and memory items of the MMSE, ACE, WMS-R; alternative images for the Boston Naming Test; alternative commands for the Token test; alternative semantic ("fruits and animals") and phonological ("letter F") categories for verbal fluency; and alternatively distributed items in the Trail Making Tests (sometimes referred to as Parts C and D). In order to ensure that differences between pre- and post-operative results were not related to potential differences in the demand of alternative stimuli, a counterbalanced design was used (five patients received one version preoperatively and the other five patients received the alternative versions preoperatively, and viceversa for postoperative assessment). Preliminary analysis showed no significant differences existed between both groups of the counterbalanced design, reason why all patients were considered as part of the same group.

Table 1
Preoperative and postoperative Mean (SD) performance and range on tests of the neuropsychological battery. Wilcoxon signed ranks $Z$ and associated $P$ values are also reported

| Neuropsychological test | Preoperative | | Postoperative | | $Z$ | $P$ |
|---|---|---|---|---|---|---|
| | Mean (SD) | Range | Mean (SD) | Range | $-0.89$ | n.s. |
| MMSE | 24.4 (5.5) | 12–29 | 27.0 (2.7) | 20- 30 | $-1.34$ | n.s. |
| ACE Total Score | 75.4 (20.2) | 27–93 | 83.5 (13.9) | 45–93 | $-0.53$ | n.s. |
| Orientation | 8.80 (1.7) | 5–10 | 9.30 (1.6) | 5–10 | $-1.72$ | n.s. |
| Attention | 6.50 (1.8) | 3–8 | 7.40 (0.7) | 6–8 | $-0.77$ | n.s. |
| Memory | 23.4 (9.6) | 0–32 | 26.1 (7.8) | 5–32 | $-0.24$ | n.s. |
| Fluency | 7.40 (3.8) | 0–12 | 10.9 (4.3) | 0–16 | $-0.24$ | n.s. |
| Language | 26.1 (3.0) | 18–28 | 26.9 (1.2) | 25–28 | $-0.54$ | n.s. |
| Visuospatial | 3.30 (1.6) | 1–5 | 4.2 (0.9) | 3–5 | $-1.64$ | n.s. |
| Boston Naming Test | 18.1 (1.4) | 16–20 | 19.4 (0.7) | 18–20 | $-2.57$ | **0.010** |
| Token Test | 19.2 (5.3) | 8–26 | 22.1 (3.5) | 13–26 | $-1.26$ | n.s. |
| Semantic fluency (animals) | 9.7 (5.7) | 0–17 | 11.5 (4.4) | 1–16 | $-0.67$ | n.s. |
| Forward Digit Span | 5.2 (0.9) | 4–7 | 5.6 (0.8) | 4–7 | $-1.08$ | n.s. |
| Trail Making Test A | 105.4 (73) | 37–280 | 86.4 (76) | 36–301 | $-0.66$ | n.s. |
| Backward Digit Span | 3.10 (0.7) | 2–4 | 3.70 (0.5) | 3–4 | $-1.94$ | **0.040** |
| Trail Making Test B | 184.7 (20) | 150–210 | 126.3 (76) | 70–270 | $-2.09$ | **0.037** |
| Phonological fluency (“P”) | 8.4 (4.7) | 0–13 | 10.9 (4.2) | 0–16 | $-2.38$ | **0.017** |
| FAB Total Score | 14.0 (5.2) | 3–18 | 15.4 (3.1) | 7–18 | $-0.30$ | n.s. |

The abbreviated assessment conducted following CSF drainage in order to determine candidacy for surgery included the AI, the ACE, and the I-QOL.

### 2.3. Statistical analysis

Wilcoxon signed-rank tests for paired samples were used to compare performance on cognitive tests before and after shunt surgery.

## 3. Results

Patients (7 males, 3 females) were 69.4 ($SD = 9.3$) years old on average, and had completed 12.9 ($SD = 3.8$) years of formal education. Table 1 summarizes group performance on each test before and after shunt surgery. Significant differences were found between basal and follow-up performances only on the Boston Naming Test ($Z = -2.57$, $p = 0.1$), the digits backwards span ($Z = -1.94$, $p = 0.04$), Part B of the Trail Making Test ($Z = -2.09$, $p = 0.037$), and the number of words produced on the phonological fluency task ($Z = -2.38$, $p = 0.017$). While slight improvements were observed for all tasks, no other significant differences were found.

## 4. Discussion

Patients included in this study had shown a positive response to continuous slow lumbar drainage of CSF and were therefore eligible candidates for VP shunting. This is, to the best of our knowledge, the first study to find a clear pattern of significant differences between preoperative and postoperative performance on tests of executive function. While slight improvements were found on all tests included in our neuropsychological battery, no other significant differences were found, with the exception of a language test, the Boston Naming Task [24].

Evidence of executive function recovery in VP shunted patients comes from three out of four tests of executive function included in our battery. First, the high demand of an executive component in the backward digit span has been well studied in the literature and more recently confirmed in patients with traumatic brain injury [29]. Accordingly, significant improvement was found on the backward version of this task, but not on the forward digit span task, considered a task of pure attention that does not rely on an executive central. Second, cognitive flexibility and set shifting tend to be impaired in the early stages of many forms of dementia, probably due to the loss of executive control. Significant improvement was found for Part B of the Trail Making Test [30], but again, the “attentional” counterpart, i.e. Trail Making Test Part A, did not show significant improvement.

Third, the fact that significant improvement was found on the phonological but not on the semantic fluency task is indicative of cognitive recovery specific to executive functioning. Usually, two types of fluency tasks are used, in which patients are asked to elicit as many words as possible in a fixed time frame, according to a phonological criterion (in our case, words starting with letter "P") or a semantic criterion (in this study, animals) [5]. Both tasks require access to lexical memory and retrieval of lexical items, but search strategies associated with each task help differentiate their executive demand. Phonological fluency requires inhibition of an ordinary strategy in which retrieval of words memory is done according to meaning [31]. Instead, semantic fluency takes advantage of this strategy, requiring only a prior retrieval of conceptual knowledge. Because inhibition is fundamentally associated with executive functions [32], phonological fluency is considered a good measure of this form of mental process. Moreover, lesion studies have shown that phonological fluency is specifically mediated by the dorsolateral prefrontal cortex [33], which has been also targeted as the neural substrate for executive functions for over four decades [34].

Our fourth test of executive function was the FAB, designed as a brief tool to assess frontal functions in patients through six subtests (abstract reasoning, mental flexibility, motor programming, sensitivity to interference, inhibitory control, and environmental autonomy) [28]. We did not find significant improvement on the total FAB score nor on any of its six subscales. While the FAB may be useful in examining frontal signs of different pathologies, several studies have reported that it is cannot be used to fully differentiate dysexecutive functions in different types of dementia such as Alzheimer Disease (AD) and the frontal variant of Frontotemporal Dementia (fvFTD) [35,36]. For this reason, the FAB may not be sensitive enough to detect executive function changes in shunted patients.

There are some limitations to this study. First, the small sample size allows for little generalization, and the study must be replicated in larger samples. However, like in works published by other authors [16], the small sample size of this study is powered by the very selective inclusion criteria. Second, because time for assessment at bedside is limited, only a restricted set of tests for each cognitive domain was included in the battery for this study. However, the significant improvement found almost exclusively on tests of executive function is worthy of further research employing both tests of executive function and tests assessing other domains, in order to determine the extent and specificity of cognitive improvements. We also suggest the inclusion of more "ecological" tests designed to detect real-life executive deficits among these patients, in order to understand the extent of impairment, and potential recovery, of activities of daily living. Third, while all patients in this study qualitatively improved their gait and incontinence following surgery, future studies should examine the relationship between cognitive amelioration and gait and incontinence improvement in depth.

The preliminary results of this study are important for several reasons. First, as already mentioned, executive functions are essential for effective performance in our complex world and have implications for real-life functioning. Being able to reverse the executive dysfunction with VP shunt surgery adds support to a crucial treatment option, targeted at improving the quality of life of patients with NPH. Second, the reversibility of executive function is in itself an intriguing process worth investigating, and may help us understand the way in which CSF may alter structures in the prefrontal cortex. As well, it may contribute to elucidate the process by which subcortical build-up of CSF may consequently damage the frontosubcortical connections. Third, this study supports the close correlation established between positive response to continuous slow lumbar CSF drainage and good prognosis of cognitive recovery. Finally, our results highlight the importance of thorough neuropsychological assessment as part of the clinical diagnosis of NPH.

## 5. Conclusions

In conclusion, our study reveals that patients with NPH who respond positively to continuous slow lumbar CSF drainage and receive a VP shunt implant, improve their performance on tasks of executive function. The high demand of this form of mental processing in real-life complex scenarios and the severe executive deficits present in both demented and non-demented NPH patients, makes this study worth replicating at a larger scale. We also encourage the inclusion of more "ecological" tests to detect real-life deficits in this clinical group.

## Acknowledgements

This study was funded by a FINECO grant.
